# Recent advances in the treatment and prevention of venous thromboembolism in cancer patients: role of the direct oral anticoagulants and their unique challenges

**DOI:** 10.12688/f1000research.18673.1

**Published:** 2019-06-27

**Authors:** Dominique Farge, Corinne Frere

**Affiliations:** 1 Université de Paris, IRSL, Paris, France; 2 Assistance Publique Hôpitaux de Paris, Saint-Louis Hospital, Internal Medicine, Autoimmune and Vascular Disease Unit, Paris, France; 3 McGill University, Montral, QC, Canada; 4 Sorbonne Université, INSERM UMRS_1166, Institute of Cardiometabolism And Nutrition, Paris, France; 5 Assistance Publique Hôpitaux de Paris, Pitié-Salpêtrière Hospital, Department of Haematology, Paris, France

**Keywords:** venous thromboembolism, cancer, low molecular weight heparin, direct oral anticoagulant

## Abstract

Venous thromboembolism (VTE) is a common complication in patients with cancer and is associated with poor prognosis. Low-molecular-weight heparins (LMWHs) are the standard of care for the treatment of cancer-associated thrombosis. Primary VTE prophylaxis with LMWH is recommended after cancer surgery and in hospitalized patients with reduced mobility. However, owing to wide variations in VTE and bleeding risk, based on disease stage, anti-cancer treatments, and individual patient characteristics, routine primary prophylaxis is not recommended in ambulatory cancer patients undergoing chemotherapy. Efforts are under way to validate risk assessment models that will help identify those patients in whom the benefits of primary prophylaxis will outweigh the risks. In recent months, long-awaited dedicated clinical trials assessing the direct oral anticoagulants (DOACs) in patients with cancer have reported promising results. In comparison with the LMWHs, the DOACs were reported to be non-inferior to prevent VTE recurrence. However, there was an increased risk of bleeding, particularly in gastrointestinal cancers. Safe and optimal treatment with the DOACs in the patient with cancer will require vigilant patient selection based on patient characteristics, co-morbidities, and the potential for drug–drug interactions.

## Introduction

Data from large registries indicate that the risk of developing venous thromboembolism (VTE) is four- to seven-fold greater for cancer patients compared with non-cancer patients, and the incidence of cancer-associated thrombosis (CAT) is increasing worldwide ^1– 5^. As many as half of CAT cases are incidentally detected ^6^. Multiple factors contribute to the risk of CAT; these include cancer type and stage, surgery, medical anti-cancer therapies, central venous catheter use, treatments for co-morbidities, and individual patient characteristics, such as extremes in body weight, malnutrition, and advanced age ^7^. Thromboembolism is the second leading cause of death after cancer progression in this patient population ^2^. CAT is a marker of poor prognosis; cancer patients who develop VTE are six times less likely to survive than cancer patients without VTE ^1^. Management of VTE with anticoagulant therapies in patients with cancer is challenging because these patients are more likely to experience VTE recurrence and major bleeding events on anticoagulants relative to non-cancer patients ^8^, which may be explained by expression of fibrinolytic factors by malignant cells, disseminated intravascular coagulation and thrombocytopenia, vascular invasion, and certain anti-cancer therapies ^9, 10^.

Low-molecular-weight heparins (LMWHs) have been recommended over vitamin K antagonists (VKAs) as the first-line treatment for CAT for over 15 years ^11^ on the basis of three positive (CANTHANOX ^12^, CLOT ^13^, and LITE ^14^) out of five (CANTHANOX ^12^, CLOT ^13^, LITE ^14^, ONCENOX ^15^, and CATCH ^16^) randomized controlled trials (RCTs) which demonstrated that LMWHs are more effective and at least as safe as VKAs. LMWHs are recommended for at least 3 to 6 months for the treatment of established VTE ^17– 22^. Primary VTE prophylaxis is recommended for cancer surgery and hospitalized patients with reduced mobility but is not recommended routinely in ambulatory cancer patients receiving systemic anti-cancer treatments.

Despite being the best anticoagulant option in patients with cancer, the use of a parenteral anticoagulant is sometimes a barrier to treatment and may result in low treatment compliance. In addition, the cost of LMWH treatment is prohibitive in certain health-care systems. In recent years, direct oral anticoagulants (DOACs) have become an alternative to LMWH for the treatment of CAT. Like LMWH, DOACs have a rapid onset of action with predictable pharmacodynamics, but they have the convenience of oral administration. Initial evidence supporting the use of DOACs in the treatment of CAT was provided by analyses of cancer patient subgroups (3–9%) ^23– 29^ included in the landmark DOAC trials, which were conducted in the general patient population ^30– 35^. In the past two years, dedicated head-to-head trials in the cancer patient population have been published with promising results ^36– 40^. Although DOACs offer certain advantages over parenteral anticoagulants, they present their own set of unique treatment challenges that require appropriate patient selection for their use. Here, we summarize the new evidence on DOAC anticoagulant therapy in cancer patients and still-unanswered questions and controversies in the field.

## New evidence for the use of DOACs in patients with cancer

### Treatment and secondary prophylaxis of established cancer-associated VTE

Results from three dedicated RCTs assessing the safety and efficacy of the DOACs in the treatment of CAT patients compared with LMWH have recently become available ^36– 38^.

The patients included in these trials reflect a more representative cross-section of the overall cancer patient population; more than half of participants had a metastatic disease (53.0–65.5%) and more than two thirds were receiving systemic anti-cancer therapy (70.0–74.0%). All studies excluded patients with an ECOG (Eastern Cooperative Oncology Group) score of more than 2. The rates of VTE recurrence and major bleeding in the LMWH groups across DOAC studies are consistent with the rates documented in the landmark trials which demonstrated the superiority of LMWH over VKA (CANTHANOX ^12^, CLOT ^13^, and LITE ^14^) to prevent VTE recurrence in cancer patients with no increase in bleeding.

The Hokusai-VTE ^36^ and SELECT-D ^37^ trials compared the safety and efficacy of DOAC and LMWH in the treatment of established CAT ( Table 1). In Hokusai-VTE ^36^, 1050 cancer patients with symptomatic or incidentally diagnosed VTE were randomly assigned to either edoxaban (60 mg daily after at least 5 days of LMWH therapy) or dalteparin (200 IU/kg daily for 1 month followed by 150 IU/kg daily) for 6 to 12 months. Edoxaban was non-inferior to LMWH in the composite primary outcome measure of recurrent VTE or major bleeding within 12 months after randomization regardless of treatment duration (hazard ratio [HR] 0.97, 95% confidence interval [CI] 0.70–1.36, *P* = 0.006 for non-inferiority, *P* = 0.87 for superiority). An analysis of the components of the primary outcome measure demonstrated that VTE recurrence rates were numerically lower with edoxaban, but that this difference was not statistically significant (7.9% [edoxaban] versus 11.3% [dalteparin], *P* = 0.09). Major bleeding was more common with edoxaban (6.9% versus 4.0%, *P* = 0.04), whereas rates of clinically relevant non-major bleeding and mortality were similar between groups. The higher rate of major bleeding in the edoxaban group was driven by gastrointestinal (GI) bleeding in patients with GI cancer. In Hokusai-VTE, patients were excluded if the need for several P-glycoprotein (P-gp) inhibitors, such as ritonavir, nelfinavir, indinavir, or saquinavir, was anticipated. Systemic use of other P-gp inhibitors, namely etoconazole, itraconazole, erythromycin, azithromycin, or clarithromycin, was not permitted at inclusion but was permitted if needed during the study with appropriate dose adjustments of edoxaban.

**Table 1.** Randomized clinical trials assessing the efficacy and safety of direct oral anticoagulants in the treatment of cancer-associated thrombosis.

Randomized clinical trials	Hokusai-VTE cancer ^36^	SELECT-D ^37^	ADAM-VTE ^38^
Number of randomly assigned patients	1050	406	300
Trial design	Non-inferiority	Pilot	Superiority
DOAC	Edoxaban: Dalteparin for at least 5 days followed by edoxaban 60 mg once daily for 6 to 12 months	Rivaroxaban: 15 mg twice daily for 3 weeks followed by 20 mg once daily for 2 to 6 months	Apixaban: 10 mg twice daily for 7 days followed by 5 mg twice daily for 6 months
Comparator	LMWH: Daltaparin 200 IU/kg once daily first 30 days followed by 150 IU/kg daily	LMWH: Daltaparin 200 IU/kg once daily first 30 days followed by 150 IU/kg daily	LMWH: Daltaparin 200 IU/kg once daily first 30 days followed by 150 IU/kg daily
Primary outcome measures	Composite measure of recurrent VTE or major bleeding within 12 months after randomization	VTE recurrence in the 6 months after randomization	Major bleeding including fatal bleeding
Primary outcome results	• Edoxaban: 12.8% • Dalteparin: 13.5%	• Rivaroxaban: 4% • Dalteparin: 11%	• Apixaban: 0% • Dalteparin: 2.1%
Major secondary outcomes	Recurrent VTE • Edoxaban: 7.9% • Dalteparin: 11.3% Major bleeding • Edoxaban: 6.9% • Dalteparin: 4.0%	Major bleeding • Rivaroxaban: 13% • Dalteparin: 4% CRNMB • Rivaroxaban: 6% • Dalteparin: 4%	Recurrent VTE (DVT, PE, fatal PE) • Apixaban: 3.4% • Dalteparin: 14.1% • Major + fatal + CRNMB • Apixaban: 9.0% • Dalteparin: 9.0%

CRNMB, clinically relevant non-major bleeding; DOAC, direct oral anticoagulant; DVT, deep vein thrombosis; LMWH, low-molecular-weight heparin; PE, pulmonary embolism; VTE, venous thromboembolism.

In the SELECT-D pilot trial ^37^, 406 cancer patients with symptomatic or incidental pulmonary embolism (PE) or symptomatic lower-extremity proximal deep vein thrombosis (DVT) were randomly assigned to rivaroxaban (15 mg twice daily for 3 weeks followed by 20 mg once daily) or dalteparin (200 IU/kg daily for 1 month followed by 150 IU/kg daily). The 6-month cumulative rate of recurrent VTE after randomization was significantly lower with rivaroxaban compared with dalteparin (HR 0.43, 95% CI 0.19–0.99). Cumulative clinically relevant non-major bleeding rates were higher with rivaroxaban (HR 3.76, 95% CI 1.63–8.69), but major bleeding rates were not statistically different between the groups (6% [rivaroxaban] versus 4% [dalteparin], HR 1.83, 95% CI 0.68–4.96). Of note, during the interim analysis, the data and safety monitoring study committee determined that although the overall bleeding events did not cross the safety boundary of excess in clinically relevant bleeding, the patients with esophageal or esophagogastric junction tumors had a trend toward major bleeding. Therefore, as a precaution, such types of patients were subsequently excluded from enrollment. Six-month survival did not differ between the two treatment arms. Major bleeding occurred more frequently with rivaroxaban than with dalteparin in patients with esophageal or gastroesophageal cancer (36% [rivaroxaban] versus 11% [dalteparin]). Most major bleeding events in the rivaroxaban group were GI. Most clinically relevant non-major bleeding events occurring with rivaroxaban were GI or genitourinary. Concomitant use of the following strong cytochrome P450 3A4 inhibitors or inducers was excluded: human immunodeficiency virus protease inhibitors or systemic ketoconazole and rifampicin, carbamazepine, or phenytoin. Concomitant use of P-gp inhibitors or inducers was excluded.

Results from the ADAM trial (ClinicalTrials.gov Identifier: NCT02585713) ^38^ were presented in December 2018 at the 60th American Society of Hematology annual meeting. Cancer patients with established CAT, including upper-extremity and splanchnic vein thrombosis, were randomly assigned to either apixaban ****(10 mg twice daily for 7 days followed by 5 mg twice daily) or dalteparin (200 IU/kg daily for 1 month followed by 150 IU/kg daily) for 6 months. The primary outcome measure of major bleeding rate was similar between the two treatment groups (0 [apixaban] versus 3/142 (2.1%) [dalteparin], *P* = 0.9956). The secondary outcome of VTE recurrence rate was lower with apixaban compared with LMWH (HR 0.26, 95% CI 0.09–0.80, *P* = 0.0182). Strong CYP3A4 inducers were excluded from the study.

***Treatment satisfaction.*** Few studies have assessed the quality of life (QoL) of cancer patients with CAT treated by LMWH. The prospective TROPIQUE (n = 409 patients with cancer) ^41^ and QUAVITEC (n = 400 patients with cancer) ^42^ cohort studies reported that most patients were satisfied or very satisfied and reassured about treatment efficacy and experience with side effects under LMWH, which did not hinder QoL improvements in those who survived to 6-month follow-up. In Hokusai-VTE ^36^, treatment termination as a result of inconvenience of dosing was reported in 4% of patients on edoxaban and 14.9% of patients on dalteparin. In the ADAM trial, QoL surveys in the ADAM-VTE trial ^38^ revealed a better tolerance to apixaban compared with dalteparin. Premature discontinuation of anticoagulant treatment in the study occurred in significantly fewer patients receiving apixaban compared with dalteparin (15%; *P* = 0.0012).

In conclusion, anticoagulant therapy with the DOACs in the treatment of established CAT resulted in similar or better rates of recurrent VTE but was associated with a higher risk of bleeding, particularly in GI and genitourinary cancers. The underlying cause of the susceptibility of the GI tract to bleeding may be due to accumulation of active drug or chemotherapy toxicity ^43^. Overall, these first trials suggest a favorable risk-benefit ratio for DOACs in the treatment and secondary prevention of established CAT. However, their safe and optimal use will require appropriate patient selection and monitoring of several parameters, particularly since the theoretical risks of drug–drug interactions have not been investigated in patients with cancer.

### Primary prophylaxis of cancer-associated VTE

About 5 to 10% of ambulatory cancer patients initiating chemotherapy will develop CAT, and up to 74% of CAT cases occur in the outpatient setting ^44^. The widely varying risk of VTE and bleeding across cancer types, stages, cancer treatments, and individual patients has resulted in study findings in this patient population that have been difficult to interpret. Two large RCTs compared LMWH with placebo in patients with different cancer types and found a significant reduction in the relative risk of VTE but with a small difference in the respective absolute risk ^45, 46^. A recent systematic review and meta-analysis reported that primary prophylaxis with LMWH compared with no treatment in all cancers reduced the rate of VTE, while significantly increasing the risk of major bleeding ^47^. However, the number needed to treat was 30, supporting previous conclusions that primary prophylaxis should not be used across patients with cancer ^47^. Studies in pancreatic cancer which is associated with considerably high VTE risks yielded better risk-benefit ratios ^48, 49^.

Several ongoing efforts to stratify patients according to VTE risk are under way in order to identify the appropriate patients who stand to benefit from primary prophylaxis. The Khorana score ^50^, which is based on readily available clinical and laboratory parameters, was developed for ambulatory patients initiating chemotherapy and is the most widely studied risk assessment model. The model was validated in independent studies ^51^, although recent publications questioned its reproducibility in certain patient populations ^52– 54^. Several modifications of the Khorana score have been proposed to improve its predictive strength and are currently being studied, including the extended Vienna Cancer and Thrombosis Study (CATS) score ^51^ and the Protecht score ^55^, as well as a simplified clinical prediction model incorporating only tumor-site category and D-dimer ^56^. The recent DOAC trials assessing the safety and efficacy of DOACs for the primary prophylaxis VTE in ambulatory cancer patients initiating chemotherapy aimed to address this issue by stratifying patients according to VTE risk ( Table 2) ^39, 40^. The studies used the Khorana score ^50^, which continues to be the most widely accepted VTE risk assessment model in this cancer patient population.

**Table 2.** Randomized clinical trials assessing the efficacy and safety of direct oral anticoagulants in the prophylaxis of cancer-associated thrombosis.

Randomized clinical trials	CASSINI ^39^	AVERT ^40^
Number of randomly assigned patients	841	574
Trial design	Superiority	Superiority
DOAC	Rivaroxaban: 10 mg once daily	Apixaban: 2.5 mg twice daily
Comparator	Placebo	Placebo
Primary outcome measures	• Composite measure of DVT, PE, and VTE-related death • Major bleeding	• Objectively documented VTE (proximal DVT and PE) over a 6-month follow-up period • Major bleeding
Primary outcome results	Composite on-treatment • Rivaroxaban: 2.6% • Placebo: 6.4% Composite – up to 6 months • Rivaroxaban: 6.0% • Placebo: 8.8% Major bleeding • Rivaroxaban: 2.0% • Placebo: 1.0%	VTE • Apixaban: 4.2% • Placebo: 10.2% Major bleeding • Apixaban: 3.5% • Placebo: 1.8%
Major secondary outcomes	CRNMB • Rivaroxaban: 2.7% • Placebo: 2.0%	CRNMB • Apixaban: 7.3% • Placebo: 5.5%

CRNMB, clinically relevant non-major bleeding; DOAC, direct oral anticoagulant; DVT, deep vein thrombosis; PE, pulmonary embolism; VTE, venous thromboembolism.

The CASSINI trial ^39^ assessed the safety and efficacy of rivaroxaban in ambulatory cancer patients initiating chemotherapy with intermediate to high risk of VTE (Khorana score of at least 2). Eight hundred forty-one patients were randomly assigned to either treatment with rivaroxaban (10 mg once daily) or placebo for up to 6 months. The primary outcome did not differ between the groups over the entire 6-month observation period (8.8% under placebo versus 6.0% under rivaroxaban; HR 0.66, 95% CI 0.40–1.09, *P* = 0.10). Patients who received rivaroxaban compared with those under placebo while on treatment had fewer primary outcome events, which was a composite measure of symptomatic or asymptomatic DVT or PE and VTE-related death (6.4% under placebo versus 2.6% under rivaroxaban, HR 0.40, 95% CI 0.20–0.80). ****There was no difference in major bleeding between the two groups (HR 1.96, 95% CI 0.59–6.49). Patients with primary or metastatic brain tumors were excluded from the study. The majority of enrolled patients had pancreatic (32.6%), gastric and gastroesophagus (20.9%), or lung (15.9%) cancer or lymphoma (7%).

The AVERT trial ^40^ compared primary prophylaxis with apixaban (2.5 mg twice daily) with placebo in 573 ambulatory cancer patients initiating chemotherapy for up to 6 months. The primary outcome was symptomatic and incidental VTE occurrence. Patients receiving apixaban had a lower risk of VTE occurrence compared with placebo (7.3% under placebo versus 1% under apixaban, HR 0.14, 95% CI 0.26–0.65, *P* <0.001). The risk of major bleeding was increased on apixaban (HR 2.00, 95% CI 1.01–3.95, *P* = 0.046). The majority of enrolled patients had gynecologic (25.7%), pancreatic (13.5%), or lung (10%) cancer or lymphoma (25.2%).

When results from the two studies were combined, the absolute difference in the incidence of symptomatic VTE between the DOAC and the placebo groups was 2.5% in the primary intention-to-treat analysis, which corresponds to a number needed to treat of 40 patients ^57^. Together, these DOAC studies indicate that primary prophylaxis in cancer patients with a Khorana score of at least 2 results in significantly lower rates of VTE. However, the risk of major bleeding was increased with apixaban. In both studies, patients considered to have a high bleeding risk were excluded. There was a high rate of discontinuation, in both the treatment and placebo groups.

## Unanswered questions and controversies

There are multiple disease-related factors, including drug absorption, distribution, metabolism, and elimination, that can affect anticoagulant pharmacokinetics in patients with cancer (reviewed in 58). The risks of over- or under-coagulating with DOACs, despite their predictable pharmacodynamics, as a result of renal or hepatic impairment, potential drug–drug interactions, and patient characteristics such as weight and age require vigilance (reviewed in 59, 60). Finally, the optimal treatment duration of anticoagulation after 3 to 6 months for patients with CAT remains unanswered. Table 3 summarizes the characteristics of the available DOAC.

**Table 3.** Characteristics of direct oral anticoagulants used or being investigated in patients with cancer.

	Dabigatran	Rivaroxaban	Apixaban	Edoxaban	LMWH
Target	FIIa	FXa	FXa	FXa	Indirect FXa/FIIa
Prodrug	Yes	No	No	No	No
Bioavailability	3–7%	10 mg dose: 100% 20 mg dose: 100% when taken together with food; 66% under fasting conditions Inter-individual variability: 30–40%	~50% Inter-individual variability: 30%	~62%	90–98% Subcutaneous
Renal excretion (percentage of administered dose)	5% (4% unchanged)	66% (33% unchanged)	27% (22% unchanged)	35% (24% unchanged)	High
Biliary-fecal excretion (percentage of administered dose)	95%	34%	56%	62%	Low
Metabolism	Glucuronidation	CYP3A4 (75%) > CYP2j2	CYP3A4 (50%)	Hydrolysis > CYP3A4 (27%)	Desulphation, Depolymerization (<10%)
Interaction with P-glycoprotein inducers/inhibitors	Yes	Yes	Yes	Yes	No

LMWH, low-molecular-weight heparin.

### Special considerations in patients with cancer

Vomiting and diarrhea are side effects of cancer treatment and can limit absorption. Changes related to inflammation and collection of extra-vascular fluids can affect distribution of the DOAC. Similarly, changes in body composition, such as reduced lean body mass, a common occurrence in elderly patients with cancer, reduce the distribution volume and increase the risk of acute toxicity, particularly when combined with anti-cancer therapy ^61, 62^. Decreased lean body mass can also result in an overestimation of renal function, which can put the patient at risk of over-anticoagulation ^58^. Thrombocytopenia, common in patients with cancer, is also a bleeding concern. Metabolism through the cytochrome P450 is altered in cancer, and there is large inter-individual variability in CYP3A activity in patients with cancer ^63^, further complicating attempts to predict the potential clinical impact of specific drug–drug interactions. Altered hepatic function and inflammatory responses have been proposed as an underlying mechanism for a decrease in drug metabolism through the CYP system in patients with cancer ^64, 65^. Furthermore, these patients are typically on multiple medications, increasing the risk of drug–drug interactions. Thus, anticoagulant pharmacokinetics can be widely variable across patients with cancer, rendering prediction of the dose-response relationship difficult. Overall, patients with cancer tend to be elderly, receive multiple concomitant drug therapies, and are more likely to have some degree of hepatic or renal insufficiency.

### Renal and hepatic impairment

About 60% of patients with cancer have decreased renal function ^66^ and this decline is sometimes sudden. Major or fatal bleeding can occur following a rapid decline in renal function in patients on anticoagulants. Renal and hepatic impairment will affect the plasma levels of the different DOACs on the basis of their respective mechanisms of elimination ^67^. Moderate hepatic impairment (Child–Pugh B) increases plasma levels of rivaroxaban only (127%). There are no data on severe hepatic impairment.

### Thrombocytopenia

Thrombocytopenia can develop directly from tumor invasion of bone marrow, as a result of secondary immune-mediated responses, or following myeloablative chemotherapy ^60^. In patients with cancer and thrombocytopenia, the risk of bleeding is increased. Full doses of anticoagulant can be used for the treatment of established CAT if the platelet count is above 50 G/L and bleeding is not evident; for patients with a platelet count below 50 G/L, decisions about treatment and dose should be made on a case-by-case basis ^11^ and available data do not support one management strategy over another (LMWH or DOAC).

### Drug–drug interactions

Drugs that significantly induce or inhibit CYP3A4, a member of the hepatic cytochrome P450 enzyme system, or ATP-binding cassette transporters, such as P-gp and breast cancer–resistant protein (BCRP), can change the plasma concentration of DOACs (reviewed in 59, 67, 68). Limited studies have documented the effects of co-administration of DOACs and strong inhibitors and inducers of CYP3A4 and P-gp ^67^.

All DOACs undergo an efflux transport mainly via P-gp, meditating drug absorption and excretion. Altered P-gp function is one mechanism of chemotherapy resistance, in which its activity limits absorption of the chemotherapy agent into malignant cells ^69, 70^. This pharmacodynamic profile suggests that inhibitors or inducers of P-gp can lead to over- or under-anticoagulation. However, this theoretical risk is difficult to predict given the wide inter-individual variability on P-gp activity ^71^. Rivaroxaban and apixaban are both substrates of CYP3A4 and therefore susceptible to changes in plasma concentrations when given concomitantly with drugs that induce or inhibit CYP3A4 ^67^. Rivaroxaban and apixaban are also substrates of BCRP.

For all of the DOACs, concomitant administration of rifampicin, a strong inducer of CYP3A4 and P-gp, produces clinically important decreases in DOAC plasma concentrations and increases the risk of thrombosis ^72– 74^. The product monographs for all of the DOACs indicate that concomitant administration with the CYP3A4 and P-gp inducers rifampicin, carbamazepine, and phenytoin and also phenobarbital in the case of apixaban, edoxaban, and rivaroxaban should be carried out with caution or avoided ^75– 78^.

### DOAC drug–drug interaction with cancer therapies

DOAC absorption can be altered by cancer treatments such as surgery and radiotherapy ^58^. The concomitant administration of DOACs with anti-cancer and adjunctive pharmacotherapies that are inhibitors or inducers of Pg-P and CYP3A4 poses a theoretical risk of VTE or bleeding by pushing circulating DOAC levels outside the therapeutic range. Direct studies on interactions between DOAC and anti-cancer therapies are not available. Certain chemotherapies, hormone therapies, and targeted therapies, such as tyrosine kinase inhibitors, are potent CYP inducers or inhibitors. Some anti-cancer treatments can also induce or inhibit P-gp. Potential interactions have been compiled (reviewed in 58– 60, 79) and are summarized in Table 4.

**Table 4.** Anti-cancer treatments predicted to affect direct oral anticoagulant plasma levels through moderate to strong interaction with CYP3A4 or P-glycoprotein or both.

	CYP3A4	P-glycoprotein
Inducers	Antimitotic agents: paclitaxel, ifosfamide, mitotane Tyrosine kinase inhibitors: vemurafenib, dabrafenib, vemurafenib Hormone therapy: enzalutamide Immunomodulators: dexamethasone, prednisone	Antimitotic agents: vinblastine Anthracyclines/anthracenediones: doxorubicin Tyrosine kinase inhibitors: vandetanib, sunitinib Immunomodulators: dexamethasone
Inhibitors	Tyrosine kinase inhibitors: imatinib, crizotinib, idelalisib, lapatinib, nilotinib Hormone therapy: bicalutamide, abiraterone Immunomodulators: cyclosporine	Tyrosine kinase inhibitors: imatinib, crizotinib, nilotinib, lapatinib, Hormone therapy: abiraterone, tamoxifen, enzalutamide, Immunomodulators: tacrolimus, cyclosporine,
Substrates	Antimitotic agents: paclitaxel, docetaxel, vinblastine, vincristine, vinorelbine Topoisomerase inhibitors: etoposide, irinotecan Anthracyclines/anthracenediones: doxorubicin Alkylating agents: ifosfamide, busulfan Tyrosine kinase inhibitors: crizotinib, imatinib, dasatinib, nilotinib, erlotinib, gefitinib, lapatinib, sunitinib, vandetanib Monoclonal antibodies: brentuximab Hormone therapy: abiraterone, enzalutamide, tamoxifen, flutamide Immunomodulators: cyclosporine , dexamethasone, sirolimus, everolimus, temsirolimus, tacrolimus	Antimitotic agents: paclitaxel, vinblastine, vincristine, docetaxel Antimetabolites: methotrexate Topoisomerase inhibitors: irinotecan, etoposide Anthracyclines/anthracenediones: doxorubicin, daunorubicin, idarubicin Alkylating agents: bendamustine Intercalating agents: mitomycin C Tyrosine kinase inhibitors: imatinib, nilotinib, lapatinib, crizotinib, vemurafenib Immunomodulators: cyclosporine, sirolimus, everolimus, temsirolimus, tacrolimus, dexamethasone

Adapted from ^58– 60, 68, 79^.

### Treatment duration for established CAT

Determining the optimal duration of anticoagulation in the treatment of established CAT remains an unresolved issue. RCTs that compared LMWH with VKA for the treatment of acute VTE in this setting followed the participants for only 3 to 6 months ^12– 16^. Since the risk of recurrent VTE remains high as long as the cancer is active, extended duration of anticoagulant therapy is usually proposed despite the lack of evidence from RCTs as long as specific anti-cancer treatments, classically defined by the use of anti-cancer surgery, chemotherapy, radiotherapy, and growth factors, are being delivered ^11^. Data are still insufficient to evaluate the specific VTE risk associated with long-term immunotherapy.

## Conclusions

New dedicated clinical trial evidence showing an efficacy of DOAC that is similar to or better than LMWH in the treatment and secondary prevention of CAT offers a new treatment option to patients with cancer. However, increased bleeding risks compared with LMWH, particularly in GI and genitourinary cancers, indicate a need for caution. Administering DOACs to cancer patients with CAT will necessitate individualized treatment decisions that consider patient characteristics, co-morbidities, and the potential for drug–drug interactions. Primary prophylaxis in ambulatory cancer patients initiating chemotherapy remains an area of uncertainty. The incorporation of VTE risk assessment models into clinical trial designs to stratify patients according to VTE risk may help identify cancer patients likely to benefit from primary VTE prophylaxis. The two recent trials assessing the safety and efficacy of DOACs in the prevention of VTE included patients with a Khorana score of at least 2 and reported a significant reduction in the rates of VTE, but the trial investigating apixaban reported increased bleeding risk. Future studies will continue to clarify which patients with cancer should receive primary thromboprophylaxis and the safety profiles of the different DOACs in this clinical setting.
